# Supplementary material for: A quorum-sensing regulatory cascade for siderophore-mediated iron homeostasis in Chromobacterium violaceum
Source: mSystems. 2024 Mar 19;9(4):e01397-23. doi: 10.1128/msystems.01397-23 (PMC11019928; doi:10.1128/msystems.01397-23)
Supplement: Supplemental figures and tables — Table S1, Table S3, Fig. S1, and Fig. S2. [file msystems.01397-23-s0001.pdf]

Supplemental information

Supplemental Table 1. Identification of insertion sites of transposon mutant strains screened for altered siderophore levels.

Locus of T8 insertion	Function of gene product	No. of transposon hits	Mutant strain	Predicted size, aa	Insertion site, aa	Area of siderophore halo in mm² (SD)*
---	---	---	CVNAL[a]	---	---	59,14 (5,27)
CV_0013	Hypothetical protein	1	7B94	163	66	8,04 (2,41)
CV_0025	Hypothetical protein	3	6E60, 7D65 and 7F65	173	46, 120 and 53	9,88 (2,22)
CV_0085	Carboxymuconolactone decarboxylase family protein	1	8B51	118	56	137,87 (24,71)
CV_0174	*dksA*	2	5G65 and 6G103	136	IR[b]	20,29 (5,48)
CV_0535	Hypothetical protein (*airM*)	21	1H74, 2G74, 2H74, 6H74, 7C74, 9B74, 9F74, 10H74, 12D74, 2H75, 4 E75, 6G75, 10C75, 1E76, 2B76, 2C76, 4C76, 4E77, 4H77, 5G79, 10B79 and 10D79	163	All in 19	104,5 (13,74)
**CV_0536**	**Two-component sensor histidine kinase (*airS*)**	1	9E104	458	154	125,94 (5,85)
**CV_0537**	**Probable response regulator with BetR domain (*airR*)**	2	2A44 and 1C52	300	60 and 61	110,3 (16,47)
CV_0635	Probable transcriptional regulator with PAS domain	2	10A38	257	15	140,2 (5,60)
CV_RS03 115	Cupin fold metalloprotein, WbuC family	6	4D39, 10C40, 8A57, 1A91, 4F91 and 2H92	169	4, 27, 83, 83, 84 and 153	93,65 (11,29)
CV_0773	Sugar ABC transporter substrate-binding protein	4	12F20, 6G24, 10D39 and 11E65	630	535, 138, 485 and 211	16,11 (2,13)
CV_0774	Phosphatase PAP2 family protein	10	6E41, 5H43, 12E43, 10B43,10B44,	953	74, 74, 74, 74,	12,28 (0,8)

			10D50, 12C51, 8E54, 10C54 and 11H72		74, 943 and IR	
CV_0775	Conserved hypothetical protein	4	12F21, 11H21, 12B21 and 3H22	90	IR	17,48 (5,04)
**CV_1057**	**Probable transcriptional regulator (*vitR*)**	1	1B14	87	33	180,13 (14,13)
CV_1218	Hypothetical protein	2	4H51 and 9H51	66	19 and 17	130,4 (19,96)
CV_1716	Methyl-accepting chemotaxis protein	1	11D54	693	228	151,45 (12,67)
CV_1918	Filamentous hemagglutinin N-terminal domain-containing protein	2	1E50 and 11H54	1628	507 and 507	146,4 (22,94)
CV_2228	NarK/NasA family nitrate transporter	2	4A50 and 11B54	400	111 and 111	133,0 (22,94)
CV_2600	Probable two-component sensor/regulator (*csrA*)	5	10E20, 2A26, 8G26, 7H26 and 4B27	924	304, 319, 308, 670 and 336	289,7 (11,14)
CV_3542	Nucleoside-diphosphate kinase	1	1A15	141	130	21,65 (2,03)
CV_3814	Type II secretion system protein GspD	2	12D51 and 3A54	708	302 and 302	16,25 (1,04)
CV_3972	DUF3304 domain-containing protein	1	1G96	182	144	11,88 (2,92)
CV_4019	UDP-N-acetyl-D-mannosamine dehydrogenase	1	9H61	413	341	160,62 (18,72)
CV_4023	Peptidoglycan bridge formation glycyltransferase FemA/FemB family protein	3	1A50, 4D50 and 12B104	331	30, 30 and 158	104,1 (18,01)
**CV_4090**	**LuxR family transcriptional regulator (*cviR*)**	51	12A50, 3B51, 11A54, 11F54, 1F56, 3A56, 2D56, 3E56, 4A56, 4B56, 5C56, 6D56, 6A56, 7B56, 8B56, 9F56, 9H56, 10B56, 10C56, 12H56, 1A57, 2F57, 5F57, 5C57, 7A57, 7H57, 9B57, 11D57,11F57, 11E57, 6E57,	265	124, 124 and IR	171,0 (22,85)

			1C59, 6E59, 1C59, 6E59, 7E60, 7H60, 10D61, 12A61, 7G62, 8E62, 9E62, 10C62, 5F63, 6G63, 7G63, 5H64, 6E65, 6F65, 7H65, 9E65, 10H65 and 11F65			
CV_4261	DUF4390 domain-containing protein	3	5D58, 6E58 and 8B58	184	159, 140 and 159	15,75 (3,4)

[a] Reference strain background of the transposon library; [b] Insertion in the intergenic region; * all indicated strains display a significant difference in siderophore activity ($p < 0.05$).

Supplemental Table 3. Strains and plasmids.

Strain or plasmids	Description[a]	Reference or source
**Strains**		
Escherichia coli		
DH5α	*E. coli* strain for cloning purposes.	(S1)
S17-1	*E. coli* strain for plasmid mobilization.	(S2)
BL21(DE3)	*E. coli* strain for heterologous expression of proteins.	Novagen
SM10λpir	*E. coli* strain for obtention of transposon mutants.	(S3)
Saccharomyces cerevisiae		
AH109	*S. cerevisiae* used in the double-hybrid yeast assay.	Clontech
Chromobacterium violaceum		
WT	*C. violaceum* ATCC 12472 wild-type (WT) strain with sequenced reference genome.	(S4)
CV^{NALR}	Strain with a *gyrA* spontaneous mutation, for transposon mutant selection.	(S5)
WT[pMR20]	WT control strain harboring the empty pMR20 plasmid.	This work.
WT[pSEVA]	WT control strain harboring the empty pSEVA plasmid.	This work.
airS::T8	CVNAL::IS*lacZ*/hah mutant strain with random insertion of T8 transposon in the *airS* (CV_0536) gene.	This work.
Δ*airS*	WT strain with *airS* gene deleted.	This work.
Δ*airS*[*airMS*]	*airS* mutant complemented with WT copy of *airMS* in the pSEVA vector.	This work.

airR::T8	CVNAL::IS*lacZ*/hah mutant strain with random insertion of T8 transposon in the *airR* (CV_0537) gene.	This work.
Δ*airR*	WT strain with *airR* gene deleted.	This work.
Δ*airR*[*airR*]	Δ*airR* mutant complemented with WT copy of *airR* in the pSEVA vector.	This work.
Δ*airSR*	WT strain with *airSR* genes deleted.	This work.
Δ*airSR*[*airMS*]	Δ*airSR* double mutant complemented with WT copy of *airMS* in the pSEVA vector.	This work.
Δ*airSR*[*airR*]	Δ*airSR* double mutant complemented with WT copy of CV_0537 in the pSEVA vector.	This work.
vitR::T8	CVNAL::IS*lacZ*/hah mutant strain with random insertion of T8 transposon in the *vitR* (CV_1057) gene.	This work.
Δ*vitR*	WT strain with *vitR* gene deleted.	This work.
Δ*vitR*[pMR20]	Δ*vitR* mutant with empty pMR20 plasmid.	This work.
Δ*vitR*[*vitR*]	Δ*vitR* mutant complemented with WT copy of *vitR* in the pMR20 vector.	This work.
Δ*vioS*	WT strain with *vioS* (CV_1055) gene deleted.	This work.
Δ*vitR*/*vioS*	WT strain with *vitR* and *vioS* genes deleted.	This work.
Δ*vioS*[*vioS*]	Δ*vioS* mutant complemented with WT copy of *vioS* in the pMR20 vector.	This work.
Δ*vitR*/*vioS*[*vioS*]	Δ*vitR*/*vioS* double mutant complemented with WT copy of *vioS* in the pMR20 vector.	This work.
Δ*fur*	WT strain with *fur* gene deleted.	(S5)
cviR::T8	CVNAL::IS*lacZ*/hah mutant strain with random insertion of T8 transposon in the *cviR* gene.	This work.
Δ*cviR*	WT strain with *cviR* (CV_4090) gene deleted.	(S6)
Δ*cviR*[*cviR*]	Δ*cviR* mutant complemented with WT copy of *cviR* in the pSEVA vector.	Lab collection.
Δ*cviR*/*cbaF*::pNPT	Δ*cviR* mutant with an insertion mutation in the *cbaF* gene.	This work.
Δ*cviR*/*vbaF*::pNPT	Δ*cviR* mutant with an insertion mutation in the *vbaF* gene.	This work.
Δ*cviI*	WT strain with *cviI* (CV_4091) gene deleted.	(S7)
Δ*cviI*[*cviI*]	Δ*cviI* mutant complemented with WT copy of *cviI*.	Lab collection.
**Plasmids**		
pNPTS138	Suicide vector containing oriT, *sacB*; Kan^R	M.R.K. Alley
pMR20	Broad-host-range low-copy vector containing oriT, Tet^R	(S8)
pSEVA	Broad-host-range low-copy vector, Kan^R	(S9)
pET15b	Expression of proteins with N-terminal His-tag; Amp^R	Novagen

pIT2	Plasmid harboring T8 transposon (IS*lacZ*/hah); AmpR,Tet^R	(S3)
pGEM-T easy	Cloning plasmid; Amp^R	Promega
pGADT7	Expression of proteins fused to GAL4 activation domain for the Double-Hybrid assay; Amp^R, *LEU2* nutritional marker for selection in yeast.	Clontech
pGBKT7	Expression of proteins fused to GAL4 DNA binding domain for the Double-Hybrid assay; Kan^R, *TRP1* nutritional marker for selection in yeast.	Clontech
pRK*lacZ*290	pRK2-derived vector with promoterless *lacZ* gene, Tet^R	(S10)
pLAC::P-*vitR*	pRK*lacZ*290 with the promoter region of *vitR* gene.	This work.
pLAC::P-*vioS*	pRK*lacZ*290 with the promoter region of *vioS* gene.	This work.
pLAC::P-*cviR*	pRK*lacZ*290 with the promoter region of *cviR* gene.	This work.

[a] Abbreviations: Kan, kanamycin; Tet, tetracycline; Amp, ampicillin; NAL, Nalidixic Acid; R, resistance.

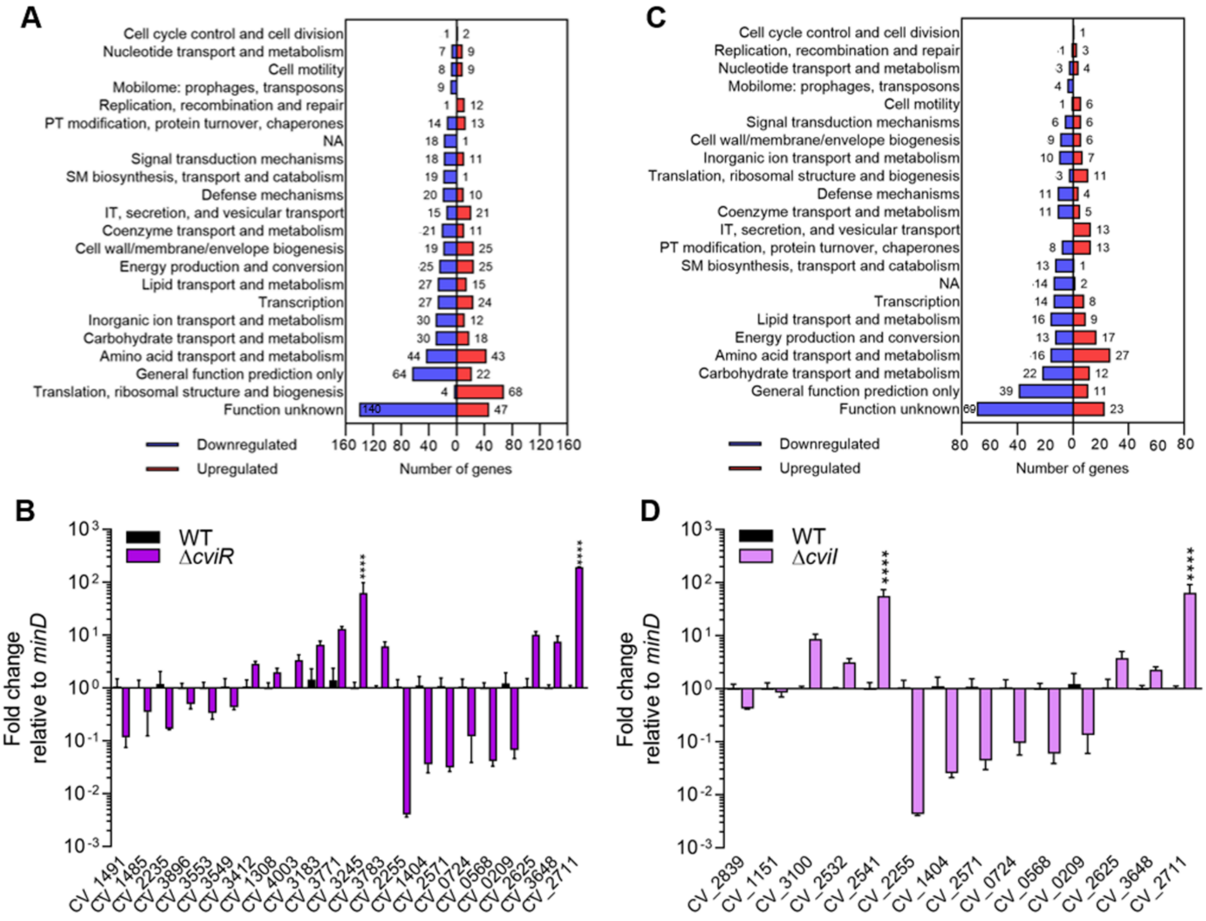

Supplemental Fig 1. Validation of differentially expressed genes in Δ*cviR* and Δ*cviI* by RT-qPCR. A. Functional categorization of genes with altered expression in Δ*cviR*. Abbreviations: NA, no annotation. IT, intracellular trafficking. PT, posttranslational. SM, secondary metabolites. **B.** Validation of differentially expressed genes in Δ*cviR.* **C.** Functional categorization of genes present in the CviI regulon. Abbreviations: NA, no annotation. IT, intracellular trafficking. PT, posttranslational. SM, secondary metabolites. **D.** Validation of differentially expressed genes in Δ*cviI*. **B and D.** cDNA was reverse transcribed from total RNA harvested from the WT, Δ*cviI*, and Δ*cviR* strains grown at high cell density (OD_{600} ~ 4.0). Expression of the indicated genes are shown as the fold change relative to WT normalized by the endogenous control *minD*. ****, $p < 0.0001$; when not indicated, not statistically significant. Two-way ANOVA followed by Tukey's multiple comparison test.

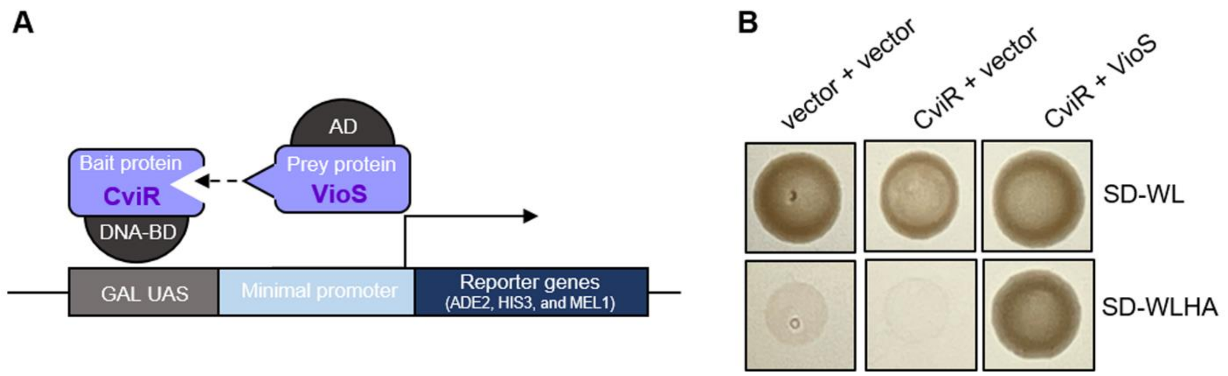

Supplemental Fig 2. VioS interacts directly with the CviR protein. A. Scheme showing the mechanism of the Two-Hybrid System (Y2H) in *Saccharomyces cerevisiae*. Two proteins of interest (CviR and VioS) are fused with two different domains of Gal4. The bait protein is fused to the DNA-binding domain of Gal4 (CviR) and the tethered protein is fused to the transcriptional activation domain of Gal4 (VioS). In yeast AH109, transcriptional activation of the reporters (ADE2, HIS3 and MEL1) only occurs in the cell if the bait interacts with the prey, leading to the activation of the GAL UAS promoter by the Gal4 transcription factor. **B.** Two-hybrid assay indicating CviR-VioS interaction. The coding regions of the *vioS* and *cviR* genes were cloned to generate fusion with the activating domain and the DNA-binding domain of Gal4, respectively. The vectors with cloning or empty were inserted into the S. cerevisiae strain AH109 through transformation. Colonies selected in SD-WL medium (Synthetic Dextrose Minimal medium, without addition of tryptophan and leucine) were then tested in SD-WLHA medium (Synthetic Dextrose Minimal medium, without addition of tryptophan, leucine, adenine and histidine) to verify the interaction between bait and prey. The empty vector was used as a negative control.

References

S1. Hanahan D. 1983. Studies on transformation of *Escherichia coli* with plasmids. J Mol Biol 166, 557-580. 10.1016/s0022-2836(83)80284-8.

S2. Simon R, Priefer U, Pühler A. 1983. A Broad Host Range Mobilization System for In Vivo Genetic Engineering: Transposon Mutagenesis in Gram Negative Bacteria. Bio/Technology *1*, 784-791. 10.1038/nbt1183-784.

S3. Jacobs MA, Alwood A, Thaipisuttikul I, Spencer D, Haugen E, Ernst S, Will O, Kaul R, Raymond C, Levy R, et al. 2003. Comprehensive transposon mutant library of *Pseudomonas aeruginosa*. Proc Natl Acad Sci U S A 100, 14339-14344. 10.1073/pnas.2036282100.

S4. The complete genome sequence of *Chromobacterium violaceum* reveals remarkable and

exploitable bacterial adaptability. 2003. Proc Natl Acad Sci U S A 100, 11660-11665. 10.1073/pnas.1832124100.

S5. Santos RERS, Batista BB, da Silva Neto JF. 2020. Ferric Uptake Regulator Fur coordinates siderophore production and defense against iron toxicity and oxidative stress and contributes to virulence in *Chromobacterium violaceum*. Appl Environ Microbiol 86. 10.1128/aem.01620-20.

S6. Batista JH, Leal FC, Fukuda TTH, Alcoforado Diniz J, Almeida F, Pupo MT, da Silva Neto JF. 2020. Interplay between two quorum sensing-regulated pathways, violacein biosynthesis and VacJ/Yrb, dictates outer membrane vesicle biogenesis in *Chromobacterium violaceum*. Environ Microbiol 22, 2432-2442. 10.1111/1462-2920.15033.

S7. Barroso KCM, Previato-Mello M, Batista BB, Batista JH, da Silva Neto JF. 2018. EmrR-Dependent Upregulation of the Efflux Pump EmrCAB Contributes to Antibiotic Resistance in *Chromobacterium violaceum*. Front Microbiol 9, 2756. 10.3389/fmicb.2018.02756.

S8. Roberts RC, Toochinda C, Avedissian M, Baldini RL, Gomes SL, Shapiro L. 1996. Identification of a *Caulobacter crescentus* operon encoding *hrcA*, involved in negatively regulating heat-inducible transcription, and the chaperone gene *grpE*. J Bacteriol 178, 1829-1841. 10.1128/jb.178.7.1829-1841.1996.

S9. Silva-Rocha R, Martínez-García E, Calles B, Chavarría M, Arce-Rodríguez A, de Las Heras A, Páez-Espino AD, Durante-Rodríguez G, Kim J, Nikel PI, et al. 2013. The Standard European Vector Architecture (SEVA): a coherent platform for the analysis and deployment of complex prokaryotic phenotypes. Nucleic Acids Res 41, D666-675. 10.1093/nar/gks1119.

S10. Gober JW, Shapiro L. 1992. A developmentally regulated *Caulobacter* flagellar promoter is activated by 3' enhancer and IHF binding elements. Mol Biol Cell 3, 913-926. 10.1091/mbc.3.8.913.
